# Clinical Characteristics, Prognostic Factors, and Outcomes of Patients With Medullary Thyroid Carcinoma: A Retrospective Review From a Tertiary Care Hospital

**DOI:** 10.7759/cureus.105353

**Published:** 2026-03-17

**Authors:** Sidra Aslam, Waqas Shafiq, Ahmed Imran Siddiqi, Anum Khan

**Affiliations:** 1 Department of Endocrinology, Shaukat Khanum Memorial Cancer Hospital and Research Centre, Lahore, PAK; 2 Department of Endocrinology and Diabetes, Shaukat Khanum Memorial Cancer Hospital and Research Centre, Lahore, PAK; 3 Department of Internal Medicine, Shaukat Khanum Memorial Cancer Hospital and Research Centre, Lahore, PAK

**Keywords:** biomarkers, carcinoma, prognosis, thyroid, treatment

## Abstract

Introduction: Medullary thyroid carcinoma (MTC) is an uncommon neuroendocrine tumor of the thyroid with a highly variable clinical course. Epidemiological and outcome data on MTC from South Asian countries are limited. This study was conducted to assess the clinical features, treatment approaches, and outcomes of patients with MTC managed at a tertiary care hospital in Pakistan.

Methods: We performed a retrospective observational study including adult patients with histologically confirmed MTC treated between December 2008 and December 2019. Data collected included demographics, tumor characteristics, multiple endocrine neoplasia type 2 (MEN2) status, biochemical markers, treatment modalities, and survival outcomes. Postoperative serum calcitonin and carcinoembryonic antigen (CEA) levels, along with calcitonin doubling time (DT), were evaluated as prognostic indicators. Statistical analyses were performed using Statistical Package for the Social Sciences version 26.0 (IBM Corp., Armonk, NY).

Results: We found a total of 90 patients having MTC during our study period, as the prevalence is scarce, so all the patients found were included (mean age = 39.3 ± 12.3 years (17-67); 43.3% male). MEN2A-associated disease was present in 13.3%. Cervical lymph node involvement and distant metastases were observed in 37.8% and 13.3% of patients, respectively. Disease-free status strongly correlated with improved overall survival (p < 0.001). Elevated post-treatment calcitonin and CEA levels, as well as shorter calcitonin DT, were associated with poorer survival. Patients with distant metastases, particularly bone involvement, had significantly worse outcomes (p < 0.001).

Conclusion: Early disease detection, achievement of disease-free status, and favorable postoperative biochemical markers are key predictors of survival in MTC. These findings underscore the importance of timely diagnosis and comprehensive management.

## Introduction

Medullary thyroid carcinoma (MTC) is a rare neuroendocrine malignancy arising from the parafollicular C cells of the thyroid gland and accounts for approximately 3%-5% of all thyroid cancers [1,2]. Unlike differentiated thyroid carcinomas, MTC does not originate from follicular cells and therefore does not concentrate radioactive iodine, rendering radioactive iodine therapy ineffective for its management [1]. The clinical course of MTC is heterogeneous, ranging from indolent disease to aggressive forms with early regional and distant metastases [2].

MTC occurs either sporadically or as part of inherited cancer syndromes, most notably multiple endocrine neoplasia type 2 (MEN2), which includes MEN2A, MEN2B, and familial MTC. These hereditary forms are caused by germline mutations in the RET proto-oncogene and account for approximately 20%-25% of all MTC cases [3]. Patients with hereditary MTC often present at a younger age and may have synchronous or metachronous pheochromocytoma and hyperparathyroidism, particularly in MEN2A [3,4]. Early identification through genetic screening enables prophylactic or early surgical intervention, significantly improving outcomes [1,5].

Surgical resection remains the cornerstone of treatment for MTC, with total thyroidectomy and appropriate lymph node dissection offering the best chance for cure [1,2]. Postoperative serum calcitonin and carcinoembryonic antigen (CEA) levels are critical biochemical markers for assessing residual disease, recurrence, and long-term prognosis [6,7]. In addition, calcitonin doubling time (DT) has emerged as a powerful prognostic indicator, with shorter DTs associated with disease progression and poorer survival [7,8].

Despite advances in surgical techniques and biochemical monitoring, long-term outcomes of MTC remain variable and are heavily influenced by disease stage at diagnosis, presence of distant metastases, and completeness of surgical resection [9,10]. Data on MTC from South Asia and other low- and middle-income countries are limited, and most existing evidence is derived from Western populations. Given regional differences in access to genetic testing, referral patterns, and disease presentation, local data are essential to better understand disease characteristics and outcomes in this population.

The present study aims to evaluate the clinical characteristics, management strategies, biochemical outcomes, and survival of patients with MTC treated at a tertiary care referral center in Pakistan, with a particular emphasis on prognostic factors influencing disease-free and overall survival (OA).

## Materials and methods

Study design and setting

This retrospective cohort study was conducted at the Department of Endocrinology, Shaukat Khanum Memorial Cancer Hospital and Research Centre (SKMCH & RC).

Ethical approval

The study was approved by the institutional review board of SKMCH & RC (IRB# EX-03-11-23-01). A waiver of informed consent was granted due to the retrospective nature of the study and use of deidentified data.

Study population

Patients aged ≥16 years with histopathological confirmation of MTC at the institutional laboratory were included. Patients with differentiated thyroid carcinoma were excluded.

Data collection

In this retrospective study, all consecutive patients diagnosed with MTC between December 2008 and December 2019 were included. Relevant clinical, pathological, and biochemical data were systematically extracted from electronic medical records, and patients were followed up for a mean duration of five years from diagnosis. Data collection was standardized using a structured questionnaire to ensure accuracy and completeness (see the Appendix). Biochemical cure refers to the postoperative undetectable serum calcitonin (<5 pg/mL) measured at six-month follow-up after surgery [1,4]. Radiological cure is the absence of residual or recurrent disease on neck ultrasonography at approximately six months postoperatively [1]. Disease-free status refers to the absence of biochemical and structural disease during follow-up at six months [1,2,4]. Persistent disease refers to the elevated serum calcitonin (>5 pg/mL) and/or radiological evidence of disease at six months after surgery [1,4]. Recurrent disease is the reappearance of biochemical and/or structural disease after an initial disease-free state [1,4]. Multiple neuroendocrine syndrome (MEN2) is an autosomal-dominant RET mutation syndrome that causes MTC, pheochromocytoma, and/or hyperparathyroidism; diagnosis is supported by family history with one or more tumors or two or more characteristic tumors, with MEN2B also showing mucosal neuromas and marfanoid habitus [1]. Calcitonin DT refers to serial postoperative serum calcitonin measurements obtained at three- to six-month intervals beginning six months after surgery. Calcitonin DT was calculated using the American Thyroid Association calculator and categorized as positive, negative, or infinite [1]. A minimum of four calcitonin measurements over two years is recommended, using the same laboratory and assay. Declining calcitonin or CEA values may produce negative DTs.

Outcomes

OS is the duration from the date of diagnosis or treatment until death from any cause [1]. Recurrence-free survival (RFS) is the time from disease-free status to detection of recurrence [1,4].

Statistical analysis

All analyses were performed using Statistical Package for the Social Sciences version 26.0 (IBM Corp., Armonk, NY). Continuous variables were reported as mean ± standard deviation, and categorical variables as frequencies and percentages using chi-square or Fisher’s exact test, as appropriate. Disease outcomes were analyzed using Kaplan-Meier survival curves, and recurrence was defined based on rising tumor markers or radiological evidence. Multivariable Cox regression was not performed due to the limited number of events, which could have led to overfitting and unstable hazard ratio estimates.

## Results

A total of 90 patients were included in the analysis. Most patients were from Pakistan (93.3%), with smaller numbers from Afghanistan (5.6%) and Iran (1.1%). OS appeared similar across all three countries; a formal statistical comparison was not performed due to small subgroup sizes.

Baseline characteristics

Cervical lymph node involvement was present in 34 patients, and the majority of patients (63.3%) had no lymph node enlargement at presentation, indicating that a significant proportion were diagnosed before extensive nodal spread (Table 1). Distant metastasis was observed in 11 patients (12.2%), with bone (5.6%), liver (5.6%), and lung (1.1%) being the most common sites. Of the 23 patients diagnosed before 30 years of age, nine (39.1%) had MEN2-associated disease. Notably, three-quarters of all MEN2 cases (9/12, 75.0%) were diagnosed before the age of 30 years, highlighting the younger age at presentation in hereditary MTC. Age distribution was comparable between surviving and deceased patients, with mean ages of 38.5 ± 12.3 and 41.4 ± 11.9 years, respectively (mean difference: -2.92 years, 95% CI: -8.98 to 3.14; p = 0.341). Levene’s test confirmed equal variances (F = 0.076, p = 0.784).

**Table 1:** Baseline characteristics ^*^Data were available for a subset of patients, and frequency was calculated accordingly SD: standard deviation; MEN2: multiple endocrine neoplasia type 2

Characteristic	Value
Age at diagnosis, mean ± SD (range)	39.3 ± 12.3 years (17-67)
Male sex, n (%)	39 (43.3%)
Country of origin, n (%)	
Pakistan	84 (93.3%)
Afghanistan	5 (5.6%)
Iran	1 (1.1%)
MEN2-associated disease, n (%)	12 (13.3%)
Positive family history (MEN2A), n (%)	3 (3.3%)
Tumor size, mean ± SD (cm)	3.9 ± 1.5
Cervical lymph node involvement, n (%)	34 (37.8%)
Unilateral neck	21 (23.3%)
Bilateral neck	9 (10.0%)
Neck + mediastinal	3 (3.3%)
No nodal involvement	57 (63.3%)
Distant metastasis at diagnosis, n (%)	11 (12.2%)
Bone	5 (5.6%)
Liver	5 (5.6%)
Lung	1 (1.1%)
None	79 (87.8%)
Extrathyroidal extension^*^, n (%)	7/74 (9.5%)
Capsular invasion^*^, n (%)	11/76 (14.5%)
Vascular invasion^*^, n (%)	11/74 (14.9%)

Clinicopathologic features and OS

In patients with the clear resection-margin group, 47 patients (83.9%) were alive, and nine (16.1%) were deceased at the last follow-up. Similarly, among patients with involved resection margins, 17 (81.0%) were alive, and four (19.0%) had died. OS proportions were comparable between these patients. Similarly, OS was comparable between patient groups with and without capsular invasion: 90.9% of patients with capsular invasion and 83.1% of those without capsular invasion were alive at last follow-up (p = 0.510). Vascular invasion was assessed in 74 patients and was present in 11 (14.9%), while 63 (85.1%) had no vascular invasion. OS was similar between the groups, with 90.9% of patients with vascular invasion and 84.1% of those without vascular invasion alive at last follow-up. Statistical analysis confirmed no significant association between vascular invasion and OS (Pearson χ² = 0.34, p = 0.56; Fisher’s exact test p = 1.00). Extrathyroidal extension (ETE) was present in seven of 74 patients (9.5%), while 67 (90.5%) had no ETE; OS was similar between groups, and there was no significant association between ETE and survival (Pearson χ² = 0.002, p = 0.964; Fisher’s exact test p = 1.00).

Distant metastasis was present in 12 of 90 patients (13.3%), most commonly affecting bone (5.6%), liver (5.6%), and lung (2.2%). OS was markedly lower in patients with metastasis, with 85.9% of patients without metastasis alive compared with 40% alive among those with lung, bone, or liver involvement. There was a significant association between distant metastasis and OS (p < 0.001). Among the 90 patients, external beam radiotherapy (XRT) was offered to 21, 20 (22.2%) received thyroid-directed XRT, and one (1.1%) received XRT to bone. Survival was higher in the no-XRT group, with 84.1% alive vs. 55.0% in the thyroid-directed XRT group; the single patient treated for bone metastasis was deceased. Overall, a higher proportion of deaths was observed among patients who received XRT compared to those who did not; however, this finding l likely reflects treatment selection in higher-risk disease rather than a direct treatment effect.

A total of five patients (5.6%) with symptomatic/progressive disease were advised tyrosine kinase inhibitors (TKIs). OS was lower in these patients, with 60% deceased compared with 21.4% deceased among patients who did not receive TKIs. There was a borderline statistically significant association between TKI therapy and OS (Pearson χ² = 3.89, p = 0.048), likely reflecting treatment of higher risk or progressive disease.

Analysis of prognostic markers

Posttreatment CEA and calcitonin levels differed significantly by OS. Because Levene’s test indicated unequal variances for both markers (CEA p < 0.001; calcitonin p < 0.001), results should be interpreted cautiously. Under the equal-variance assumption, mean posttreatment CEA was significantly lower in alive patients than in those who died (mean difference = -486.8 ng/mL, p < 0.001), and mean posttreatment calcitonin was also significantly lower among survivors (mean difference = -6,143.4 pg/mL, p = 0.002). These findings suggest that markedly elevated posttreatment tumor markers are associated with worse survival outcomes in MTC.

Calcitonin DT data were available for 85 patients. Of these, 13 patients (15.3%) had a positive DT, 26 (30.6%) had a negative DT, and 46 (54.1%) had an infinite DT. Among the 56 patients with complete data, recurrence was strongly associated with calcitonin DT category. Recurrence occurred in six of seven patients (85.7%) with positive DT, two of six patients (33.3%) with negative DT, and in none of the 43 patients (0%) with infinite DT. Conversely, all patients with infinite DT remained recurrence-free during follow-up. This association between calcitonin DT and recurrence was highly statistically significant (Pearson χ², p < 0.001; Fisher’s exact test, p < 0.001).

Outcomes

**Figure 1:**
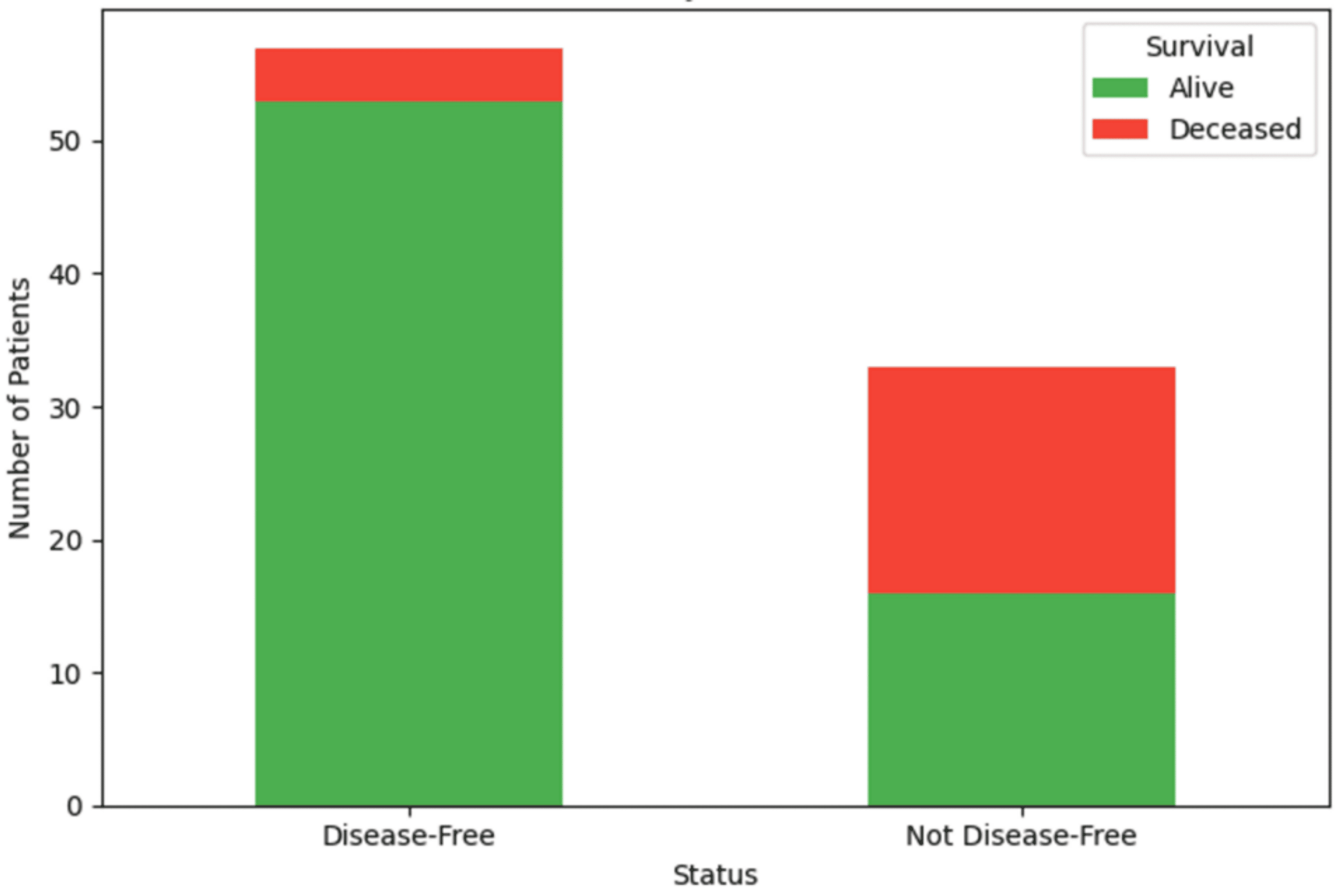
Disease-free status and overall survival

Among the 90 patients analyzed, 57 patients (63.3%) became disease-free on follow-up after surgery, while 33 patients (36.7%) had persistent disease (Figure 1). In contrast, among patients who were not disease-free, only 16 (48.5%) were alive, while 17 (51.5%) had died at the last follow-up. A highly significant association was observed between disease-free status and OS (p < 0.001).

Among 90 patients, 21 deaths (23.3%) occurred over a maximum follow-up of 175 months, while 69 patients (76.7%) were censored at last follow-up. The estimated OS was 90.6% at 12 months, 80.2% at five years, and 68% at 10 years. Median OS was not reached. The estimated mean OS time was 130.1 months (standard error (SE) = 7.42; 95% CI: 115.6-144.7), representing a restricted mean survival estimate due to right-censoring at the longest observed follow-up time. SEs widened at later time points due to decreasing numbers at risk.

Among 57 patients who achieved initial disease control, nine recurrences (15.8%) were observed over 156 months, while the remaining 48 patients were censored without evidence of recurrence. Estimated RFS was 98.2% at 12 months, 84.3% at five years, and 75.6% at 96 months. Median RFS was not reached, and the survival curve plateaued beyond 96 months with no additional recurrences.

## Discussion

This study presents a large single-center experience of MTC from a tertiary care hospital in Pakistan and provides important insights into disease characteristics, management strategies, and outcomes in a South Asian population. With 90 patients included, this cohort represents one of the larger institutional series reported from the region and adds to the limited body of literature from low- and middle-income countries.

The mean age at diagnosis in our cohort was 39.3 years, which is younger than that reported in most Western series, where the mean age typically ranges between 45 and 55 years [2]. This difference is largely attributable to the substantial proportion of hereditary disease in our population, as three-quarters of MEN2-associated cases were diagnosed before the age of 30 years. Similar age distributions in hereditary MTC have been reported in international registries and large multicenter studies, emphasizing the strong association between RET mutations and early disease onset [3,11].

Cervical lymph node involvement was present in 37.8% of patients at diagnosis, while 63.3% had no nodal disease. This rate is lower than that reported in several Western cohorts, where nodal metastases at presentation have been observed in 50%-70% of cases [9,10,12]. The relatively lower nodal burden in our cohort may suggest earlier diagnosis in a subset of patients or referral bias to a high-volume endocrine surgery center. Nonetheless, lymph node involvement remains a well-established predictor of persistent disease and biochemical recurrence in MTC [13].

Distant metastases were identified in approximately 13% of patients, most commonly involving bone and liver, followed by lung. These findings are consistent with previously published series that have demonstrated bone and liver as the most frequent metastatic sites in MTC [10]. Importantly, metastatic disease was strongly associated with inferior OS in our cohort, with survival dropping to 40% among patients with distant metastases. Similar survival disparities have been reported by Roman et al. and Raue and Frank-Raue, who demonstrated significantly reduced long-term survival in patients with metastatic MTC, particularly those with pulmonary involvement [14].

Achievement of disease-free status following initial surgery emerged as one of the strongest predictors of survival. Nearly two-thirds of patients became disease-free postoperatively, although recurrence occurred in 17.5% during follow-up. Patients with persistent disease had markedly worse outcomes, a finding that aligns with prior studies showing that biochemical persistence after surgery is associated with disease progression and reduced survival [13,15]. These observations reinforce the importance of complete initial surgical resection performed at experienced centers.

The five-year OS in our cohort was 80%, slightly lower but comparable to international data from the Surveillance, Epidemiology, and End Results Program, which report five-year survival rates of 85%-90% for MTC. Large international series, including studies by Roman et al., have similarly reported five-year survival rates exceeding 85% in predominantly early-stage disease [2,10]. The five-year RFS was 84.2%, indicating effective early disease control. The modest survival difference may reflect referral bias, advanced-stage presentation, and resource limitations in a tertiary-care setting. Long-term estimates should be interpreted cautiously due to small numbers at risk.

Posttreatment calcitonin and CEA levels differed significantly between surviving and deceased patients. Elevated postoperative tumor markers have consistently been associated with residual disease and adverse outcomes in MTC [6,7,16]. Furthermore, calcitonin DT demonstrated a strong association with recurrence in our cohort. Notably, none of the patients with infinite DT experienced recurrence, while the majority of patients with positive DTs developed recurrent disease. This finding mirrors those reported by Barbet et al. and Meijer et al., who identified calcitonin DT as one of the most powerful prognostic indicators in MTC [7,8].

Traditional histopathological features such as capsular invasion, vascular invasion, and ETE were infrequent in this cohort and were not significantly associated with OS. While these features are generally regarded as markers of aggressive disease, their prognostic value has been variable across studies and may be outweighed by factors such as metastatic spread and biochemical persistence [10,13].

Patients who received XRT or kinase inhibitors (TKIs) demonstrated lower OS. However, this finding likely reflects treatment selection bias, as these modalities were reserved for patients with advanced, progressive, or metastatic disease. Large trials and real-world studies have shown that TKIs such as vandetanib and cabozantinib improve progression-free survival but have not consistently demonstrated an OS benefit, particularly in heavily pretreated or advanced cases [17,18].

This study is limited by its single-center retrospective design, the nonavailability of genetic testing, missing data for certain pathological variables, and the variable follow-up pattern of our patients. Additionally, formal comparisons between subgroups from different countries were limited by small sample sizes. Given the limited number of recurrence events (n = 9), multivariable Cox regression modeling would be statistically constrained and prone to overfitting; therefore, survival outcomes were primarily analyzed using Kaplan-Meier methodology. Despite these limitations, the study provides valuable region-specific data and highlights key prognostic factors relevant to clinical practice in resource-limited settings.

## Conclusions

Our findings confirm that early-stage disease, absence of distant metastases, achievement of disease-free status after surgery, low postoperative tumor marker levels, and favorable calcitonin DT are associated with positive survival outcomes in MTC. These results emphasize the need for early diagnosis, access to genetic screening, expert surgical management, and rigorous biochemical surveillance to improve outcomes in patients with MTC.

## Appendices

**Table 2:** Questionnaire for data collection MEN2: multiple endocrine neoplasia type 2; MTC: medullary thyroid carcinoma; CEA: carcinoembryonic antigen; XRT: external beam radiotherapy; TKI: tyrosine kinase inhibitor

S. no.	Question	Data
1.	Diagnosis date	-
2.	Age	-
3.	Gender	Male Female
4.	Province	-
5.	Country	Pakistan Afghanistan Iran
6.	MEN2 syndrome	Yes No
7.	Family history of MTC	Yes No
8.	Largest thyroid nodule size radiologically (cm)	-
9.	Nodal disease	Unilateral cervical Bilateral cervical Mediastinal None
10.	Distant metastasis	Lungs Liver Bone None
11.	Resectability	Yes No
12.	Surgery type	Total TT ± unilateral or bilateral neck dissection
13.	Tumor focus	Unifocal Bifocal
14.	Tumor size (cm) post-surgery	-
15.	Margins	Cleared Involved
16.	Capsular invasion	Yes No
17.	Vascular invasion	Yes No
18.	Immune stains	Amyloid Calcitonin Both None
19.	Perineural invasion	Yes No
20.	Extrathyroidal extension	Yes No
21.	Was CEA checked at diagnosis?	Yes No
22.	CEA at diagnosis (ng/mL)	-
23.	Was calcitonin checked at diagnosis?	Yes No
24.	Calcitonin at diagnosis (pg/mL)	-
25.	XRT given	If yes, mention the site
26.	TKI referral	Yes No
27.	Disease-free	If yes, mention date
28.	CEA post-treatment	-
29.	Calcitonin post-treatment	-
30.	Calcitonin doubling time	Positive negative infinity
31.	If positive - doubling time (report in months)	-
32.	Recurred	Yes No
33.	Recurrence date	-
34.	Status at 5-year follow-up	Alive dead
35.	Date of last follow-up	-
